# PROKARYO: an illustrative and interactive computational model of the lactose operon in the bacterium *Escherichia coli*

**DOI:** 10.1186/s12859-015-0720-z

**Published:** 2015-09-29

**Authors:** Afshin Esmaeili, Timothy Davison, Andrew Wu, Joenel Alcantara, Christian Jacob

**Affiliations:** 1 Department of Computer Science, Faculty of Science, University of Calgary, 2500 University Drive NW, Calgary, T2N 1N4 Canada; 2 Department of Biochemistry and Molecular Biology, Cumming School of Medicine, University of Calgary, 3330 Hospital Drive NW, Calgary, T2N 4N1 Canada; 3 Department of Microbiology, Immunology and Infectious Diseases, Cumming School of Medicine, University of Calgary, 3330 Hospital Drive NW, Calgary, T2N 4N1 Canada

**Keywords:** Computational modeling, Mathematical model, Agent-based model, E. coli simulation, Lactose operon model

## Abstract

**Background:**

We are creating software for agent-based simulation and visualization of bio-molecular processes in bacterial and eukaryotic cells. As a first example, we have built a 3-dimensional, interactive computer model of an *Escherichia coli* bacterium and its associated biomolecular processes. Our illustrative model focuses on the gene regulatory processes that control the expression of genes involved in the lactose operon. *Prokaryo*, our agent-based cell simulator, incorporates cellular structures, such as plasma membranes and cytoplasm, as well as elements of the molecular machinery, including RNA polymerase, messenger RNA, lactose permease, and ribosomes.

**Results:**

The dynamics of cellular ’agents’ are defined by their rules of interaction, implemented as finite state machines. The agents are embedded within a 3-dimensional virtual environment with simulated physical and electrochemical properties. The hybrid model is driven by a combination of (1) mathematical equations (DEQs) to capture higher-scale phenomena and (2) agent-based rules to implement localized interactions among a small number of molecular elements. Consequently, our model is able to capture phenomena across multiple spatial scales, from changing concentration gradients to one-on-one molecular interactions.

We use the classic gene regulatory mechanism of the lactose operon to demonstrate our model’s resolution, visual presentation, and real-time interactivity. Our agent-based model expands on a sophisticated mathematical *E. coli* metabolism model, through which we highlight our model’s scientific validity.

**Conclusion:**

We believe that through illustration and interactive exploratory learning a model system like *Prokaryo* can enhance the general understanding and perception of biomolecular processes. Our agent-DEQ hybrid modeling approach can also be of value to conceptualize, illustrate, and—eventually—validate cell experiments in the wet lab.

## Background

A bacterial cell, as elementary as it might be from a biological perspective, is a good model organism to study biological complexity. Illustrations and animations are powerful ways to explore and describe complex systems. David Goodsell’s book “The Machinery of Life”, in which *E. coli* bacteria and biomolecules play a prominent role, is an excellent example of how to communicate scientific concepts through textual descriptions in combination with illustrative drawings of cellular structures across a range of scales [1]. Through such illustrations, molecular and cellular structures become tangible and attain meaning within their specific metabolic contexts. This promotes a deeper scientific understanding of the systems at hand.

**Fig. 1:**
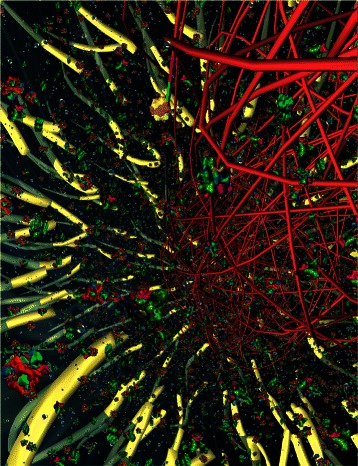
The *Prokaryo* cytoplasm. A snapshot from an interactive simulation with DNA structure, water, ribosomes, RNA polymerases, *β*-galactosidase and lactose. In this scene over 70,000 particles are rendered in realtime. Compare Fig. 11 for protein shapes and colour representations

Motivated by Goodsell’s visuals, we have taken his highly detailed, illustrative “snapshots” one step further: we bring the biomolecular interactions of a bacterial cell alive as 3-dimensional computer simulations (Fig. 1). The output from our *Prokaryo* model is similar to the animations generated through Harvard University’s BioVisions project [2]. In order to visualize the inner workings of a eukaryotic cell, Harvard BioVisions produced an eight-minute animation entitled “The Inner Life of a Cell” [3]. Unlike Goodsell’s static illustrations, BioVisions enhances the understanding of structural and cellular biology by providing movement, flow, and a sense of real dynamics. However, an important element is missing in these animations: a way to interact with and explore the models in real time.

Interactivity enables inquiry-based investigation through self-directed exploration, which is a powerful and effective way of learning [4]. This is especially true for comprehending complex system dynamics. Imagine being immersed in a bacterial cell, cruising along the cell surface, slipping through the membrane, diving into the cytoplasm, and exploring the dynamic worlds inside a cell — all under your own navigational control.

In this paper we show a first step in this direction with *Prokaryo*, an illustrative, interactive 3D model with integrated simulations of biomolecular processes inside an *E. coli* cell. Our approach combines a sophisticated mathematical model [5] and an agent-based approach to simulate regulatory processes of the lactose operon.

## Biological background: the bacterium *Escherichia coli*

Billions of bacteria live inside our intestinal tracts with an estimated 100 billion billion bacteria on Earth [6]. Due to its ease of growth and versatility as an organism, *Escherichia coli* (*E. coli*) has been the centre of many biological discoveries. With a genome that encodes for 4300 proteins, *E.coli* is one of the first organisms to have its DNA sequenced, which provides the basis for understanding the genetic programs of a bacterial cell [1].

**Fig. 2:**
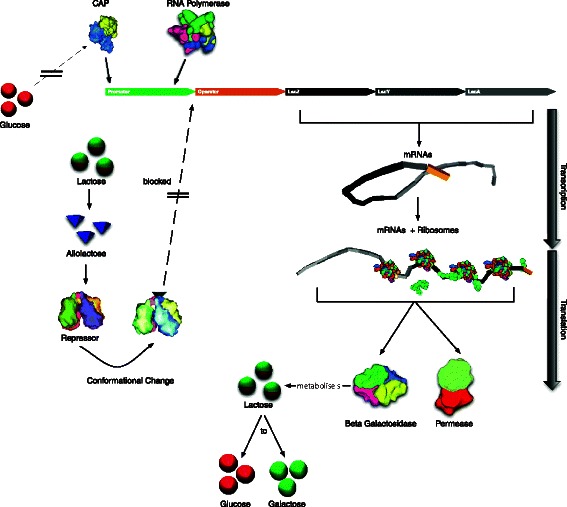
Schematic of the lac operon regulatory pathways

Surprisingly though, exact details of how a biological cell works—to a large extent—still remain a mystery. This is even the case for comparatively simple bacterial (prokaryotic) cells. Eukaryotic cells are even more complex, and consequently even harder to model [7]. A good starting point for understanding the molecular dynamics inside a bacterium is to investigate how its small building blocks (molecules) interact with other building blocks and structural elements (cytoplasm, periplasm) within the cell. For instance, proteins acting as repressors regulate gene expressions, which further trigger a cascade of events. Understanding the regulation processes is crucial for identifying cellular responses to internal and external signals. As a cell reacts to signals by switching different genes on and off, different proteins are manufactured in response [8]. Given the prominent role of gene regulation in a cell’s life cycle, we have chosen a classical, well-studied gene regulation mechanism inside *E. coli* to be modeled and simulated as part of this work: the lactose operon switch (Fig. 2).

In the following section, we explain the lactose operon switching mechanisms by using illustrations taken from our *Prokaryo* model, which we will discuss in more detail later.

### The lactose operon and the *Prokaryo* virtual cell

**Fig. 3:**
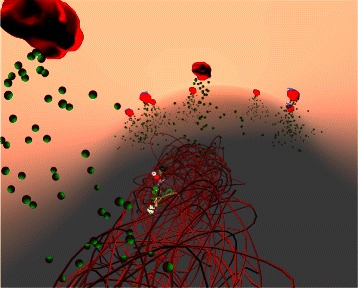
Transport of lactose as observed from the inside of the *Prokaryo* cell, within the cytoplasm. Sitting on the surface of the membrane, the inner half of the transmembrane proteins are depicted in red. Lactose molecules are shown as red and green spheres. In the centre, DNA is represented as a coiled structure. Note the exposed operon region on the DNA, where ribosomes, repressor and peptide chains are partly visible. This region is highlighted in Fig. 5

**Fig. 4:**
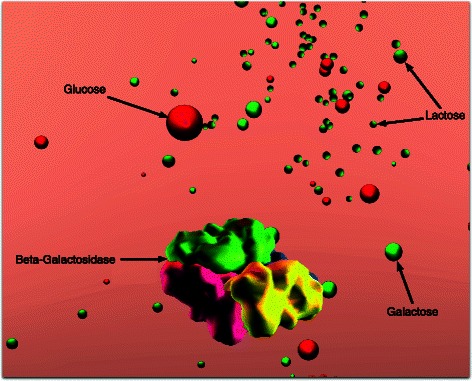
*β*-galactosidase metabolism. Upon collision, *β*-galactosidase metabolizes lactose molecules into glucose and galactose. The lactose molecules are represented as half red and half green spheres. All red spheres depict glucose molecules; green spheres represent galactose

**Fig. 5:**
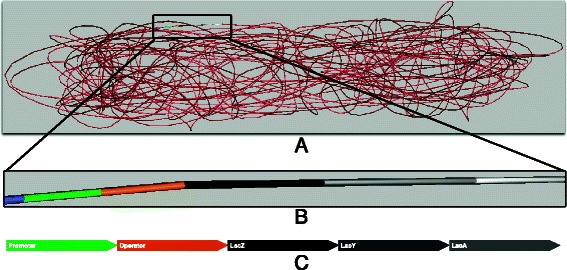
Colour coded representation of the genetic sections that comprise the lactose operon. **a** Operon location relative to the DNA structure; **b** Colour coding of gene segments used in the simulation; **c** corresponding labels of gene segments

**Fig. 6:**
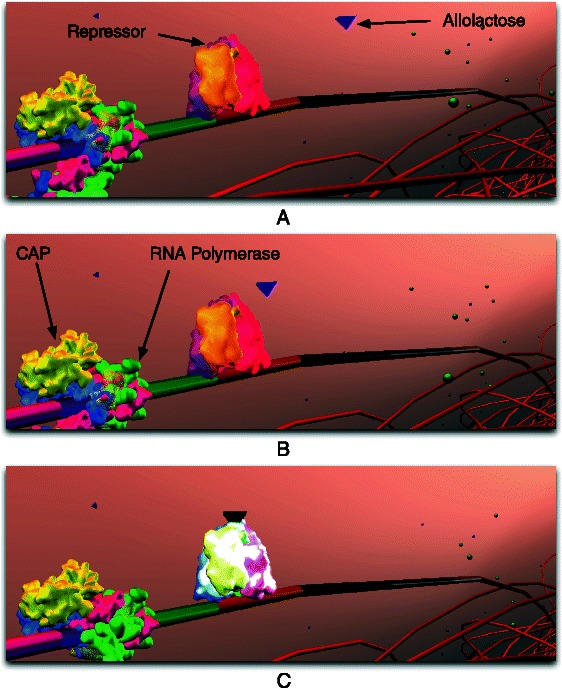
Repressor Inactivation. **a** and **b** RNA polymerase and CAP are blocked by the repressor. Allolactose approaches repressor. **c** Allolactose has docked onto repressor, causing its conformational change (visualized by a colour change of the repressor protein). Subsequently, repressor will undock from DNA, initiating transcription (Fig. 7)

Half a century ago, Jacob and Monod laid the foundations of molecular biology by illustrating an example mechanism for gene regulation: the lactose operon [9]. *E. coli* thrives in a lactose-rich environment. After lactose is transported through the periplasm into the cytoplasm (Fig. 3), lactose needs to be dissociated into glucose and galactose (Fig. 4). Glucose is one of the sources of energy for the bacterium. Consequently, a protease, in this case *β*-galactosidase, needs to be expressed to perform the cutting. A more complete schematic view of the protein interactions involved in the lac operon is presented in Fig. 2. The three structural genes of the lac operon — lacZ, lacY and lacA (Fig. 5) — code for *β*-galactosidase, lactose permease and thiogalactoside transacetylase, respectively. *β*-galactosidase metabolizes lactose into glucose and galactose. Permease is a transmembrane protein necessary for lactose uptake. We ignore production of thiogalactoside transacetylase due to its lack of participation in the actual lac operon regulation [10]. The lac repressor acts as a negative regulator, which prevents RNA polymerase from transcribing when lactose levels are low. In the presence of allolactose (a bi-product of lactose metabolism) a complex forms between allolactose and the repressor. This allolactose-repressor complex causes a conformational change in the repressor and inactivates it. As a consequence, the allolactose-repressor complex is unable to bind to the operator region of the lactose promoter, thus leaving the operator site accessible for RNA polymerase (Fig. 6).

**Fig. 7:**
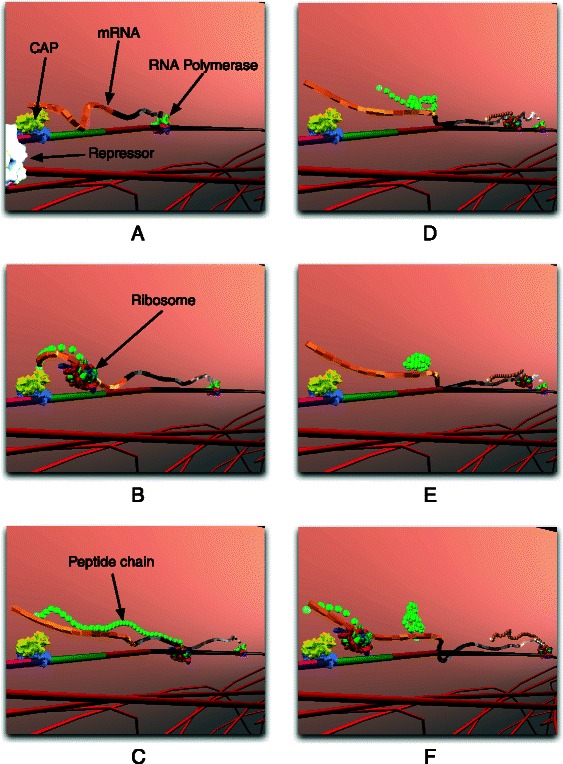
Transcription and Translation. **a** Polymerase initiates transcription and generates mRNA; (**b** and **c**) Ribosomes initiate translation on each mRNA; (**d**) Translation of mRNA generates peptide chains, which (**e**) fold and get converted to a protein structure (compare Fig. 8); (**f**) Multiple ribosomes translate mRNA at the same time

**Fig. 8:**
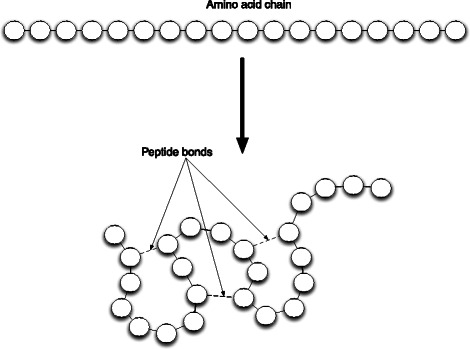
Protein Folding Heuristics: Illustration of a folding polypeptide (amino acid chain) with randomly inserted peptide bonds, which create attractive forces. Simulated physics [14] leads to the folding

The lac operon consists of three operators *O*_1_, *O*_2_ and *O*_3_. A repressor bound to *O*_1_ inhibits transcription initiation at a higher rate compared to a repressor bound to *O*_2_ and *O*_3_ which has almost no inhibitory effect [11, 12]. In this paper, we consider repressor binding to operator *O*_1_ only. This is obviously an oversimplification of the actual competitive binding events. We will demonstrate, however, that through our modular, agent-based approach one can add competitive binding into our simulation. We have captured and described competitive binding in another agent-based model of the *λ*-switch gene regulatory mechanism [13].

The second regulatory mechanism (positive regulation) in the lac operon is controlled by glucose, which is *E. coli*’s preferred carbon and energy source. As the concentration of extracellular glucose decreases, the intracellular production of cyclic AMP (cAMP) increases. cAMP binds to cAMP receptor protein (CRP) to form the cAMP-CRP complex, also known as catabolite activator protein (CAP). The CAP complex binds just upstream of the lactose promoter and assists (through positive regulation) the RNA polymerase in transcribing the lac operon (Fig. 6 b). The lac genes are regulated by CAP; this increases the affinity of RNA polymerase to transcribe the operon. Thus, the lac operon is subject to negative (repressor) and positive (CAP) regulation. At low glucose and high lactose levels, CAP is bound to the promoter, and the repressor is inactivated in the presence of allolactose.

**Fig. 9:**
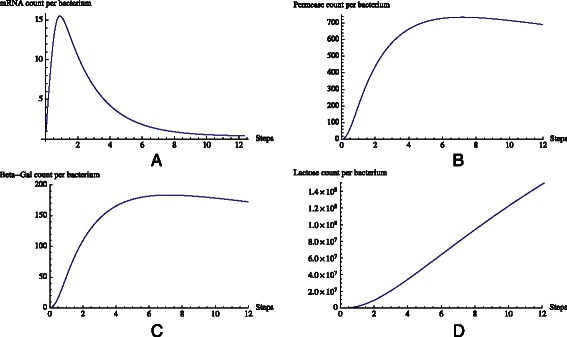
ODE model results. Quantities (in count per bacterium − cpb) of mRNA (**a**), permease (**b**), *β*-galactosidase (**c**) and lactose (**d**) as predicted by the equation set in Table 1

**Table 1:** Mathematical model to capture counts of intercellular mRNA (*M*), LacZ (*E*) and lactose (*L*) as reproduced from [27]. A summary of key symbols is listed in Table 2 with constants explained in Table 4

$\dot {M} = D k_{M} P_{D} P_{R} - \gamma _{M} M$	(5)
$\dot {E} = k_{E} M - \gamma _{E} E$	(6)
$\dot {L} = k_{L} \beta _{L} \beta _{G} Q - 2 \phi _{M} \mathcal {M} B - \gamma _{L} L$	(7)
*A*=*L*	(8)
*Q*=*E*	(9)
*B*=*E*/4	(10)
$P_{D} = \frac {p_{p} (1\, +\, p_{c} (K_{\textit {pc}}\, -\, 1))}{1\, +\, p_{p} p_{c} (k_{\textit {pc}}\, -\, 1)}$	(11)
$p_{c} = \frac {K^{n_{h}}_{G}}{K^{n_{h}}_{G}\, +\, Ge^{n_{h}}}$	(12)
$P_{R} = \frac {1}{1\, +\, \rho (A)\, +\, \frac {\xi _{123} \rho (A)}{(1\, +\, \xi _{2} \rho (A))(1\, +\, \xi _{3} \rho (A))}}$	(13)
$\rho (A) = \rho _{\textit {max}} \left (\frac {K_{A}}{K_{A}\, +\, A}\right)^{\!\!4}$	(14)
$\beta _{L} = \frac {Le}{k_{L}\, +\, Le}$	(15)
$\beta _{G} = 1 - \phi _{G} \frac {Ge}{k_{G}\, +\, Ge}$	(16)
$\mathcal {M}= \frac {L}{k_{M}\, +\, L}$	(17)

**Table 2:** Abbreviations of mathematical functions used in the equation set of Table 1

Lac operon mathematical function abbreviations	
*Ge*	Extracellular glucose
*Le*	Extracellular lactose
*A*	Intracellular allolactose
*Q*	Intracellular permease
*B*	Intracellular *β*-Galactosidase
*P*_*D*_	Negative effect of external glucose on the initiation rate of transcription (via catabolite repression)
*P*_*R*_	Probability that the lactose promoter is not repressed
*β*_*L*_	Positive effect of external lactose on its uptake rate
*β*_*G*_	Negative effect of external glucose on lactose uptake (inducer exclusion)

RNA polymerase binds to the promoter and initiates transcription to produce messenger RNA (mRNA) (Fig. 7). Ribosomes translate mRNA into peptide chains, which fold into *β*-galactosidase and permease. For folding of peptide chains, we use a simplified folding heuristic where amino acid chains are simulated with peptide bonds at random locations (Fig. 8). Subjected to simulated physics [14], the illusion of protein folding is created. Due to *Prokaryo*’s modular, component-based system architecture, the program module that encapsulates protein folding can be enhanced at a later time, once we have a more comprehensive understanding of the molecular interaction processes involved.

## Methods

Computational modeling and analysis provide insights into complex systems and can be used in education and research. Two common approaches for developing complex system abstractions are Differential Equation (DE) models [8] and multi-agent (MA) models [15]. Both modeling paradigms have their own strengths and weaknesses. The main challenge in building a bacterium model *in silico* is to capture emergent properties that none of the constituent parts alone possesses. Two general modeling approaches are explored in order to model and simulate a bacterium and to view the cellular processes from a holistic perspective. First, we use parameterized systems of DEs that describe the lac operon dynamics (Table 1). Second, a hybrid agent-based approach models the lac operon’s heterogeneous entities (agents) in an evolving network of interactions (Figs. 6 and 7).

Other whole cell in-silico simulation systems have been developed, most notably *Virtual Cell* [16] and *Smoldyn* [17]. *Virtual Cell* converts mathematical descriptions of reaction networks into differential equations handed to numerical solvers. The output is software code that can be used for further simulation analysis. *Virtual Cell* offers graphical interfaces to define reactions and to access shared simulations. In contrast to *Prokaryo*, no user interaction is possible during the simulations. *Smoldyn*, on the other hand, simulates molecules as individual units, which diffuse and react, and captures natural stochasticity in cell scale environments. Although various forms of output are possible (data files, image sequences, movies), *Smoldyn* only offers a command line interface, and is not built for realtime interaction during simulations. In contract to both *Virtual Cell* and *Smoldyn*, *Prokaryo*’s focus is on real-time interaction in simulated 3D cell spaces, with emphasis on illustrative and educational presentation of its simulations. *Prokaryo*’s code base can handle general cell simulations, such as *Virtual Cell* and *Smoldyn* but is here primarily presented as a simulator to capture molecular reactions related to the lactose operon.

### Mathematical models of the lac operon

Mathematical modeling has had enormous success in disciplines such as physics, astronomy, social sciences and engineering. Biological systems, with their high level of complexity and lack of quantitative information, have been a great challenge to model. However, with recent developments of new experimental methods in generating vast amounts of data and technological advancements in the data processing power of computers, there is a renewed interest in modeling biological systems [18]. Cellular processes such as lactose metabolism and transport can be approximated by a network of chemical reactions. First examinations of the lac operon go back to nearly half a century ago when Jacob and Monod [9] as well as Novick and Wiener [19] examined the lac operon’s induction mechanism as an on/off switch.

More recent approaches to modeling the lac operon dynamics have been reviewed by Santillan and Mackey [20], who present an in-depth overview of current advancements in the field of lac operon models. Following their review, we provide a short background on these modeling approaches.

One of the most detailed models of the lac operon, which relies heavily on experimental data, is the work of Wong et al. [21]. Based on a 13-dimensional DE system, their model includes (1) catabolite repression, where glucose represses synthesis of *β*-galactosidase and permease by inhibiting the production of cAMP, and (2) inducer exclusion, reducing the efficiency of lactose permease to transport lactose molecules into the cell.

Vilar et al. [18] suggest a simpler model by integrating different scales into their equations. They consider the molecular, cellular and population level dynamics of the lac operon, yet ignore both catabolite repression and inducer exclusion in their model.

Yildirim & Mackey [22] modeled the lac operon dynamics through a 5-dimensional equation system. The focus of Yildirim and Mackey’s work lies on the dynamics of *β*-galactosidase, permease, intercellular lactose and allolactose. Catabolite repression and inducer exclusion is ignored. This model considers delays in transcription and translation and their effects on the lac operon dynamics.

Santillan & Mackey [23] developed a model of both regulatory mechanisms of the lac operon – catabolite repression and inducer exclusion – using a 6-dimensional model, including all three operators acting on RNA polymerase to enhance transcription of the lac operon.

Van Hoek and Hogeweg [24, 25] constructed a population based mathematical model of the lac operon evolution. They investigated the lac operon switch response to lactose and artificial inducers via the introduction of stochasticity to simulating protein production in bursts.

Santillan et al. [5] investigated bistability of the lac operon gene regulatory system and validated their results with experimental data from Ozbudak et al. [26]. Santillan et al. developed a model of the lac operon system that indicated that bistability guarantees the metabolism of lactose only when the carbon source (glucose) is not available. Later, Santillan et al. [27] extended their work with a model that included variable growth rates in *E. coli*. Both models are DE-based using Gillespie’s Tau-Leap algorithm [28, 29].

The models reviewed above are based on ordinary differential equations (ODE) with chemical kinetics formalism. ODEs are only valid, however, when the molecular counts are very large, which is actually not the case for the regulatory units in the lactose operon switch [20]. The next section describes the lac operon ODE model, based on the work of Santillan et al., that we use in our *Prokaryo* system.

## The Santillan bistability model of the lac operon

Santillan et al.’s [5] model focuses on bistability of the lac operon. Tables 1 and 2 list the Santillan model equations and the functions used to describe the inter-molecular dynamics. We have chosen this model as our starting point based on its minimalistic approach and its improvements over the earlier models discussed above. The Santillan model captures mRNA (M), lacZ polypeptide (E) and internal lactose (L) concentrations.

We consider the Santillan model complementary to our agent-based modeling approach. We will use the results from the Santillan model to validate our hybrid *Prokaryo* model, which we describe in more detail in the following section.

According to Santillian’s model, the system is assumed to be glucose starved with an initial glucose concentration of 10 *μ*M. Lack of glucose promotes the formation of CAP complex which, in turn, aids RNA polymerase in transcribing the DNA. The initial external lactose concentration (*Le*) is assumed to be 1000 *μ*M. The presence of lactose and allolactose will result in a conformational change in the repressor’s structure causing it to detach from its position on the DNA (Fig. 6). The promoter is enhanced by the presence of CAP complex, becomes available, and is therefore no longer blocked by the repressor (Fig. 7). Thus, RNA polymerase begins transcription of DNA, and mRNA is generated. The resulting change in mRNA counts is illustrated in Fig. 9 a. Ribosomes translate mRNA into peptide chains, which fold into permease and *β*-galactosidase. Figure 9 b shows the average permease count per bacterium. The model assumes that an average of 750 permeases participate in transporting lactose into the cell. This causes the lactose count inside the cell to increase (Fig. 9 d).

We have implemented the Santillan model into our *Prokaryo* simulation framework using the GNU Scientific Library (GSL), an open source numerical library in C and C++ [30]. The ODEs (Table 1) were solved using the GSL differential equation solver. In Fig. 9 we summarize the resulting concentration changes of mRNA, permease, *β*-galactosidase and internal lactose, as captured by the Santillan model. These plots do coincide with the results reported in the original paper [5].

## Agent-based model of the lac operon

Although research in biological domains traditionally focuses on exploring interactions by means of equation-based modeling, agent-based models are finding their way into more computational models of biological and biomedical systems. The research community has explored agent-based modeling in areas such as cancer research [31], immunology [32], clinical studies [33], vascular modeling [34] and developmental systems [35]. Agent-based modeling (ABM), also known as *individual-based modeling* (IBM), simulates interactions of agents with each other and their environment. Local interactions among agents give rise to complex global patterns, also known as emergence [36]. These effects are usually not visible by inspecting individual agents alone: the whole — through the network of interactions — is more than the sum of its parts.

Jacob and Burleigh in 2004 [37] built a spatial three-dimensional agent-based model of the lac operon. Their model treated each protein as an autonomous agent that interacts with other agents and their environment based on physical collisions. They modeled the lac operon on a double-helix plasmid with the Watson-Crick complementary pattern that closely mimics (part of) the genetic structure of the bacterial DNA. Jacob and Burleigh’s model simulated the repressor protein and *β*-galactosidase (LacZ) effects on lac operon dynamics. However, the effect of permease (LacY), a transmembrane protein which helps to import lactose into the cell, was ignored. Jacob and Burleigh’s model also abstracts the effect of catabolite repression on the system. Rather, they focus on key gene regulatory interactions of the lac operon. With *Prokaryo*, we took a similar approach to Jacob and Burleigh’s model and built an extended 3-dimensional agent-based model of the lac operon.

### LINDSAY composer: an ABM-ODE hybrid lac operon model

Although agent-based models can replicate emergent phenomena from agent interactions, simulating collective behaviours of individual agents is not without cost. A major limiting factor is the large number of interacting agents that need to be taken into account. The number of interactions that need to be tracked increases exponentially with the number of agents. In the lac operon example, a true multi-agent model would have to capture billions of interactions. Thus, it is computationally challenging to capture all the agent interactions, and run the model in real time to offer user interaction at all times. As interactivity is one of our main objectives, we needed to find a compromise between model accuracy and model responsiveness.

One way to address the high computational cost is through differential equations, which can inform the ABM of changes in the numbers of agents over time. However, differential equations are not the best choice to capture (1) system dynamics with spatial constraints, (2) when the number of reacting agents is low and (3) when the system is sensitive to small perturbations. All these aspects play a role in most biomolecular interactions, and are particularly prominent in the lac operon regulatory system.

We added an agent-based modeling framework to the differential equation set described above as the Santillan model [5]. This hybrid modeling approach combines continuous mathematical models, to capture large scale changes in biomolecular concentrations, with discrete agent interactions among smaller numbers of entities (a few to thousands). In order to implement a cellular model that incorporates both mathematical and agent-based simulations of a bacterium, we use a physics enabled simulation platform called *LINDSAY Composer*, which we developed in our lab [38]. *Composer* combines high-end 3D graphics libraries [39] with a physics engine [14] and provides a (game) development environment for building visually appealing, semi-realistic agent-based models. Through hierarchies of interconnected ‘components’, *Composer* provides the necessary software infrastructure to build, run, and visualize customized simulations and interact with them in real time. *Composer* has been used to also build models of immune system processes [40], blood coagulation [41], and developmental systems [42]. Videos that illustrate *Composer* functionality and the graphical user interface for real-time interaction are available on the *LINDSAY* website (http://www.lindsayvirtualhuman.com/?page_id=469). The programming interface for *LINDSAY Composer* is similar to currently available game development software, such as Unity 3D (http://unity3d.com) or Unreal Engine (http://unrealengine.com).^1^

### Hybrid model architecture

The processes of transcription, translation, and protein folding are modelled through agents. Transcription and translation is performed using agents which represent RNA polymerases, mRNAs, and ribosomes. Peptide chains, which are also represented as agents, fold into proteins.

**Fig. 10:**
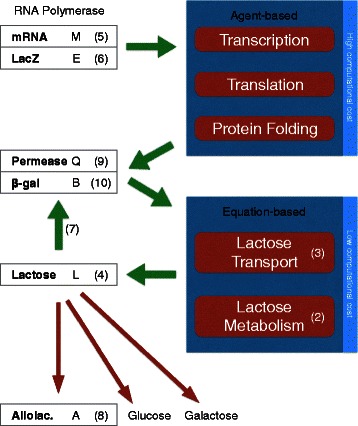
Functional architecture of the lactose operon hybrid model in *Prokaryo*. Transcription, translation and protein folding are modeled using an agent-based approach. The quantities for lactose transport into the cytoplasm and lactose metabolism within the cytoplasm are modeled through a continuous mathematical model (Table 1). The white boxes list the relevant proteins and their symbols used in the mathematical model as described in the text. The corresponding update equations are indicated by the numbers in brackets

The quantities for lactose transport into the cytoplasm and lactose metabolism are modeled using the continuous ODE system (Table 1). The numbers generated from the mathematical model, in turn, inform the display components of *Prokaryo* from which the visual representations for the agents and particles that represent lactose, glucose, and galactose are generated. This functional division of our hybrid model is illustrated further in Fig. 10.

**Fig. 11:**
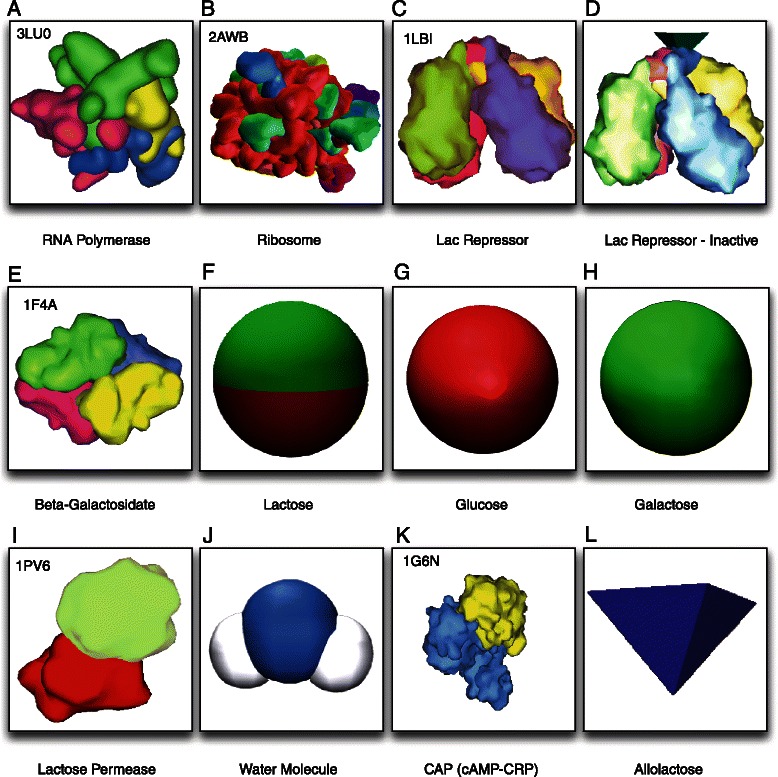
Panels of 3D shapes used for protein agents and other molecular entities in the *Prokaryo* simulation. Protein shapes are imported from the Protein Data Bank (PDB) [53]. For these, PDB IDs are depicted in the top left corner of the panel

In order to closely mimic actual protein structures within the simulated cell, we use mesh representations of the 3-dimensional protein structures from the Protein Data Bank (PDB), the world’s largest repository of protein structural data [43]. More precisely, our *Prokaryo* model employs the following entities: the *E. coli* cell, RNA polymease, lac repressor, CAP (cAMP-CRP) complex, mRNA, ribosome, permease, *β*-galactosidase, glucose, galactose, water molecules, allolactose and lactose. Figure 11 depicts the 3-dimensional representations for each of these agents.

## Coding the biomolecular agents

In this section we use pseudo-code to describe the implemented behaviour for each of our key agents in the *Prokaryo* model: repressors, RNA polymerases, messenger RNAs, ribosomes, and polypeptide chains. For the remaining sections, we do not explicitly add the term ‘agent’ any more, if the context is clear. For example, we refer to the ‘repressor agent’ simply as ‘repressor’, the ‘RNA polymerase agent’ as ‘RNA polymerase’, etc.

### Repressor: agent representation and coding

The repressor (Fig. 11 c and d) is implemented as an agent that can be in the following three states according to Algorithm 1:

- **WANDERING**: The repressor is randomly moving within the cytoplasm.
- **DOCKED**: Upon collision with the operator-promoter region, the repressor undergoes a change in state from WANDERING to DOCKED, after which it blocks RNA polymerase from transcribing the operon.
- **INACTIVE**: The repressor has undergone conformational change by the presence of allolactose attached to its active site (Fig. 11 d). The repressor will undock from its operator-promoter region and will not participate in the blocking of transcription initiation by RNA polymerase.

The conformational change of the repressor upon the docking of allolactose is depicted by a colour change (Fig. 6). Note that in Line 19 of Algorithm 1, after the repressor undocks from the promoter, it needs to move away from the promoter for at least one second so that the undocking event is recognized. This is due to the nature of the physics engine which registers rigid body collisions every 0.3 seconds, an internal parameter of the *Bullet Physics* simulation engine [14].^2^ Without an enforced one second delay, due to overlapping rigid bodies the physics engine would register another collision within 0.3 seconds after the repressor is undocked.



### RNA Polymerase: agent representation and coding

RNA polymerase (Fig. 11 a) is the agent which initiates the transcription of the lac operon regulatory mechanism (Algorithm 2). RNA polymerase moves randomly within the cytoplasmic space of the cell. Upon approaching the DNA and colliding with the promoter section of the operon, RNA polymerase attaches itself to the promoter-operator section. In an attempt to initiate transcription, RNA polymerase starts scanning the DNA codons. If a repressor is actively blocking the promoter-operator site, RNA polymerase can no longer proceed and will undock from the DNA. In case the promoter-operator region is not blocked by a repressor, RNA polymerase initiates transcription. While RNA polymerase transcribes the genes on the DNA, it releases a newly formed mRNA strand which detaches once the polymerase reaches the stop codon at the end of the operon (Fig. 7 a-f).

As outlined in Algorithm 2, RNA polymerase is an agent implemented as a finite state machine with the following internal states:

- **WANDERING**: Polymerase randomly moves inside the cytoplasm.
- **TRANSCRIBING**: Polymerase has encountered a promoter section and attaches to DNA. Transcription is initiated and polymerase can be in one of the following sub-states:
  - **ON_PROMOTER**: Polymerase is docked on the DNA and starts scanning the promoter moving toward the operator.
  - **ON_OPERATOR**: Once polymerase reaches an operator after scanning the promoter, it will change its transcribing state to ON_OPERATOR and moves along the DNA. If a repressor is docked on the operator, polymerase either undocks form the DNA or initiates transcription based on *Ta*, the transcription initiation affinity constant, 0≤*T* *a*≤1 (Table 3). In *E. coli* multiple repressors constantly dock and undock from the promoter-operator region. Between these repressor docking and undocking events, RNA polymerase might attach and initiate transcription. Before docking, each RNA agent generates a random number 0≤*τ*≤1.

**Table 3:** List of key parameters used in the *Prokaryo* model: parameter values have been set in order to replicate experimental data as reported from wet lab experiments and in the literature [1]

Parameter	Description	Value
*n*	Nucleotides per unit	3
*Tr*	Transcription speed	60 base pairs per second
*Gt*	Glucose threshold	40000 count per bacterium
*Ro*	Ribosome offset	0.1
*Tf*	Translation frequency	0.25 *s* *e* *c* ^−1^
*Rd*	Ribosome dock delay	1.0 *s* *e* *c* ^−1^
*Ptc*	Protein threshold count	3.0
*Rlt*	Repressor lactose threshold	2000 cpb
*Ta*	Transcription affinity	0.01
*St*	simulation time factor	10
    Transcription occurs for *τ*≤*T* *a*.
  - **ON_LACZ**: Polymerase starts transcribing the lacZ gene and generates an mRNA as it moves along toward the lacY gene.
  - **ON_LACY**: Polymerase has finished transcribing lacZ and is initiating transcription of lacY. mRNA elongation is continued as the polymerase scans along lacY.
  - **ON_LACA**: Polymerase successfully transcribed lacY and is initiating transcription of lacA.
- **UNDOCKING**: Either polymerase has reached the stop codon at the end of the lacA gene or has encountered a repressor at the promoter-operator region, thus detaching itself from the DNA.

One of the major challenges of simulating cell dynamics is the time mapping between *in vivo* to *silico*. To realistically model these dynamics, a scale factor is necessary to speed up slow processes or slow down fast processes. It takes 8.23 seconds (on average) for the RNA polymerase agent *in silico* to fully transcribe the lac operon. Based on the length of the lac operon (4941 base pairs [44]) and the maximal *in vivo* RNA transcriptional speed (approximately 60 base pairs per second [45]), on average it would require 82.35 (4941/60) seconds for transcription to complete. Every second in the simulation is mapped to 10 seconds *in vivo* (Table 3). Thus, the time required for the RNA agent to complete transcription is 8.23 seconds which corresponds to 82.3 seconds within a real *E. coli* bacterium [1].

### Messenger RNA: agent representation and coding

**Fig. 12:**
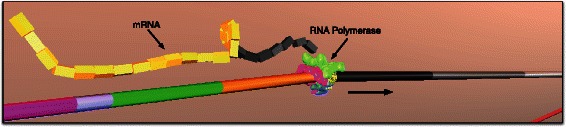
An RNA Polymerase transcribing the DNA generating an mRNA. Each colour coded section on the DNA refers to a specific gene. Direction in which transcription occurs is depicted by the arrow

**Fig. 13:**
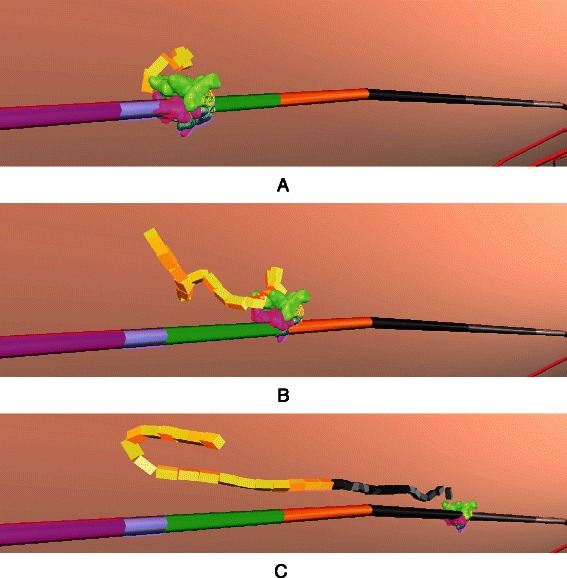
DNA transcription by RNA polymerase resulting in the generation (elongation) of messenger RNA. Transcription initiation is depicted in **a**. As the transcription progresses (**b**), mRNA elongation occurs (**c**)

**Fig. 14:**
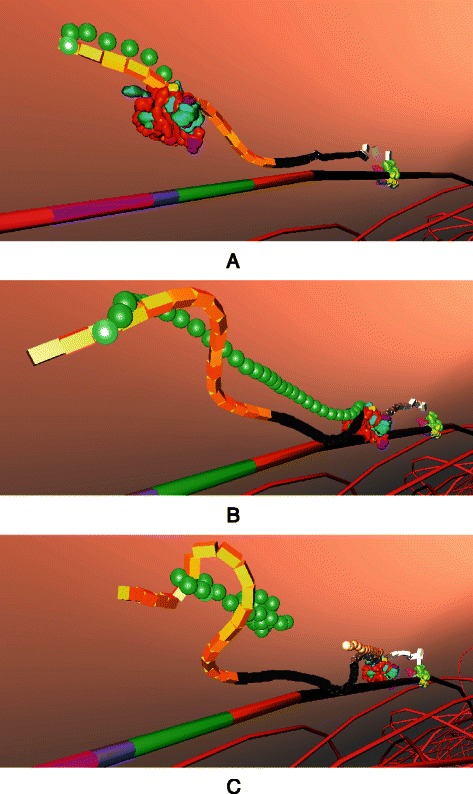
Ribosomal translation of mRNA. The ribosome scans and begins translation of the mRNA (**a**). Peptide chains are formed (**b**) and the chain begins to fold (**c**)

mRNA (Fig. 12) is an agent that does not exist at the beginning of the simulation. This is because mRNA is dynamically generated in real time upon transcription of the operon. mRNA generation is initiated by transcription of lacZ. As RNA polymerase transcribes the three genes of lac operon, mRNA elongation occurs (Figs. 13 and 14).

mRNA is treated as an agent with the following properties and states:

- *Lifetime*: upon generation of an empty mRNA by RNA polymerase, each mRNA is assigned a random lifetime between 1 to 6 minutes which is equivalent to 6 to 36 seconds in the simulation. The range is chosen to mimic mRNA lifetime in bacterial cells [46, 47].
- *Age*: is a parameter determining how long mRNA has existed within the system. Once mRNA is generated, its age is initialized to zero, *age* =0. At every simulation step, *age* is incremented by a value of *Δ*T: *age* =*age* +*Δ* *T*.^3^ This parameter is used as part of the mRNA degradation where mRNA is removed from the cell once *age* ==*lifetime*.
- *Availability State*: During mRNA generation (elongation), multiple ribosomes dock on the mRNA to initiate translation. Ribosomes can translate the mRNA simultaneously. In order to avoid collisions between translating ribosomes, there has to be a gap between translation initiation (ribosome spacing). The availability parameter enforces this gap between ribosomes docking on the mRNA and allows for a minimal number of nucleotide distance between each translating ribosome. After a ribosome has docked, the availability state of mRNA becomes UNAVAILABLE. This will block other ribosomes from docking onto the mRNA to avoid collisions between ribosomes translating the mRNA [47]. Once the current ribosome has progressed, mRNA becomes AVAILABLE for the next ribosome to initiate transcription.

In *E. coli*, ribosomes are readily available. In this study, to simplify interactions and not visually overwhelm the user with ribosomes that block visualizing the whole process, ribosome agents are created by mRNA agents. This is based on the assumption that at any point in time there are numerous ribosomes colliding with mRNA.



### Ribosome: agent representation and coding

The cell is filled with hundreds of ribosomes (Fig. 11 b) which are constantly seeking an mRNA to translate. Once a ribosome collides with a proper section of an mRNA (based on the availability state of the mRNA) it will initiate translation. As translation progresses, ribosomes use the genetic information to generate an appropriate peptide chain. Upon completion of translation, this peptide chain will fold into a protein.

Once a ribosome agent is created by an mRNA agent, it gets attached to the first codon. The ribosome then becomes active and initiates translation. It will traverse the mRNA’s codon one by one and add the appropriate amino acid sequences (based on the mRNA codon) to the peptide chain. The ribosome generates a separate peptide chain for each gene which later folds into a protein. Peptide chain elongation occurs during translation of a specific gene. The chain is detached once the ribosome moves on to the next codon corresponding to the next gene in the sequence. Once the ribosome reaches the end of an mRNA, the last polypeptide chain it has generated will detach and start folding into a protein.^4^ The ribosome agent is removed from the simulation after it has finished its mRNA transcription.

In a real cell, after translation of an mRNA strand, ribosomes become readily available for translation again. In our model, once a ribosome completes translation, it is removed from the simulation. This simplification does not affect the system due to the fact that ribosomes continuously dock and translate mRNA.





Much like the challenge presented for RNA polymerase transcription, translation by the ribosome in *E.coli* proceeds at a maximum speed of about 20 aa/sec (amino acids per second) [48]. Given that there are 4941 base pairs and every 3 base pairs code for one amino acid, the number of amino acids is 1647 (4941/3). Translating the amino acids at 20 aa/sec results in a total of 82.35 seconds to complete translation (1647/20). Again, considering the simulation time in relation to real time, 82.35 real time seconds map to 8.23 (82.35/10) seconds in the simulation. Interestingly enough, this is the same time span required for RNA polymerase to transcribe DNA.

### Polypeptide chain: agent representation and coding

As the name suggests, the polypeptide chain is composed of a chain of peptides joined together that provide a backbone for protein molecules. The peptide chains are obtained from amino acids that fold into proteins. The details of protein folding are beyond the scope of this paper. Thus, to simplify the folding process and yet mimic the structural formation of amino acids in real *E. coli*, a simplified folding mechanism of chains is introduced. Figure 14 a and b depict a ribosome translating a mRNA generating a peptide chain. Later this chain will fold into a protein (Fig. 14 c).

Once generation of a polypeptide chain is completed, the chain detaches from the ribosome. Once the chain is detached, a random number of peptide bonds (based on the length of the chain) is introduced among randomly chosen pairs of amino acids (Fig. 8).

## The *Prokaryo* E. coli cell as a hybrid model

Lac operon regulation occurs within the bacterial cytoplasm. The agents and entities involved in gene regulation (Fig. 11) interact and communicate with each other in specific regions within the cell. Inspired by Goodsell’s illustrations [1], our intention is to visualize the ‘crowdedness’ of a cell. For the actual cell body we used a custom-built, 3-dimensional mesh model of an *E. coli* bacterium cell body [49]. The *E. coli* model consists of a double layer membrane, a flagellum and pili on the surface of the outer membrane. The long, tubular structure within the cytoplasm represents the DNA as has been generated by us procedurally. The cell does not act as an agent, but rather represents a container for all agents within the cytoplasm.

**Fig. 15:**
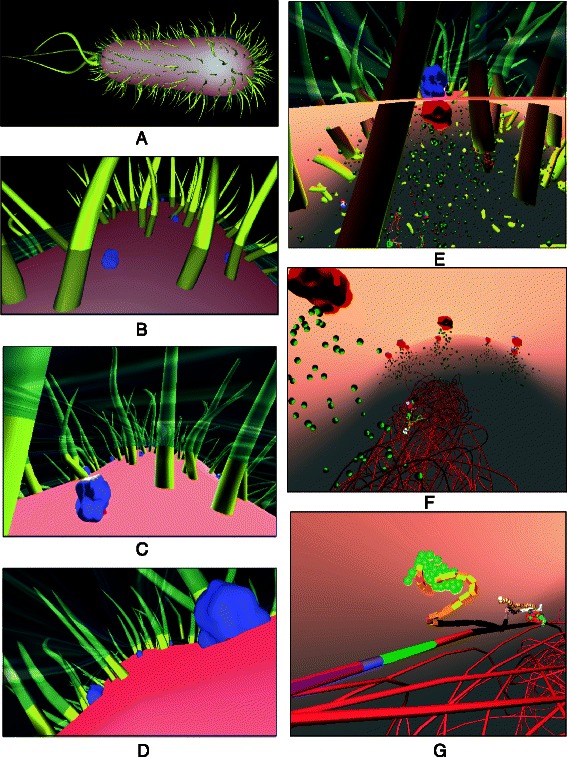
A *Prokaryo* Fly-through. The snapshots are taken while navigating through the virtual cell model. **a** approaching the cell from the outside; **b** close to the membrane, with pili and lactose permease pumps visible; **c** and **d** just below the periplasm between membranes; **e** focus on lactose permease, with lactose and other molecules in the background; **f** cytoplasmic space with coiled DNA structure in the center. **g** close-up of the lactose operon sections on the DNA with mRNA and folding amino acid chains. For this fly-through, most proteins and other entities inside the cell are not depicted. The rendering and hiding of specific molecules is controlled by the user. Hidden molecules are not visible, yet remain active elements in the simulation, as depicted in Fig. 1

The cell environment itself is divided into three subsections: the space outside (relative to the bacterium), the periplasm (between membranes) and the cytoplasm (inside the cell). Figure 15 shows screenshots of a realtime fly-through from outside the cell into the cytoplasmic space.

Occupying most of the cytoplasm, *E. coli*’s DNA is represented as a thin, tubular structure. The procedurally generated DNA structure covers an appropriate portion of the cytoplasm and is to scale relative to its *E. coli* body and its internal proteins. Instead of a double-helix, DNA is represented as a more simplistic shape, which facilitates the simulated physics calculations. On the DNA, a specific segment is chosen to represent those sections that are involved in the lac operon (Figs. 3 and 5). The section representing the operon is subdivided into colour coded segments for promoter, operator, LacZ, LacY and LacA genes, respectively.

### ABM-ODE model switching

The agent-based model captures transcription, translation and protein folding (Fig. 10).^5^ Once a peptide chain folds, the folded structure is replaced with a three-dimensional model of the respective protein: permease or *β*-galactosidase (Fig. 11). The agent model communicates and synchronizes with the mathematical equations to compute the number of agents involved in lactose metabolism based on *β*-galactosidase production and lactose transport rate, which is based on permease production. Based on the number of *β*-gal protein agents, the equation based model computes the rate at which lactose is metabolized. Similarly, permease counts are used to compute the rate at which lactose is transported into the cell.

More precisely, we increment the *β*-galactosidase count every time a corresponding peptide chain is folded. The rate *l*_*mtbol*_ of lactose being broken down per *β*-galactosidase protein is [5]:

(1) $$ l_{mtbol} = \frac{2 (\phi_{M} \times L)}{k_{M} + L}.   $$

Here, *L* represents internal lactose; *k*_*M*_ is the maximal transcription initiation rate of the promoter (2.0 *s* *e* *c* ^−1^), and *ϕ*_*M*_ is the maximum rate of lactose-to-glucose metabolism (3.6×10^4^ *m* *i* *n* ^−1^) (Table 4). Consequently, the number of lactose entities, *L*_*mtbol*_, metabolized per *Δ* *T* with *b* entities of *β*-galactosidase present is:

(2) $$ L_{mtbol} = l_{mtbol} \times b.   $$

**Table 4:** List of constants used in our reproduced model (compare Table 1)

Lactose operon model parameter list		
Parameter	Value	Description
*μ*	0.02 *m* *i* *n* ^−1^	Bacterial growth rate.
*D*	2 *m* *b* *p*	Lac promoter concentration.
*k*_*M*_	180 *m* *i* *n* ^−1^	Maximal transcription initiation rate of the lac promoter.
*k*_*E*_	18.8 *m* *i* *n* ^−1^	Translation initiation rate of lacZ transcript.
*k*_*L*_	6.0×10^4^ *m* *i* *n* ^−1^	Maximal lactose uptake rate per permease.
*γ*_*M*_	0.48 *m* *i* *n* ^−1^	lacZ mRNA dilution/degradation rate.
*γ*_*E*_	0.03 *m* *i* *n* ^−1^	Lac permease degradation/dilution rate.
*γ*_*L*_	0.02 *m* *i* *n* ^−1^	Lactose degradation/dilution rate.
*k*_*pc*_	30	Cooperative promoter-CAP binding site interaction
*p*_*p*_	0.127	Polymerase binding probability to the lac promoter.
*ϕ*_*M*_	3.6 ×10^4^ *m* *i* *n* ^−1^	Max. rate of lac-to-allolac and lac-to-gal metabolism
*K*_*G*_	2.6 *μ* *M*	CAP complex binding affinity to DNA based on external glucose concentration.
*n*_*h*_	1.3	CAP complex binding affinity to external glucose.
*ξ*_2_	0.05	Affinity of active repressor for Operator *O*_2_.
*ξ*_3_	0.01	Affinity of active repressor for Operator *O*_3_.
*ξ*_123_	163	Stability of the *O*_1_- *O*_2_- *O*_3_-repressor complex.
*ρ*_*max*_	1.3	Repression affinity of lac operon promoter
*K*_*A*_	2.92 ×10^6^ *m* *p* *b*	Allolactose-repressor subunit dissociation rate.
*k*_*L*_	680 *μ* *M*	Half-saturation constant for lactose uptake rate.
*ϕ*_*G*_	0.35	Permease activity as a function of inside glucose concentration
*k*_*G*_	1.0 *μ* *M*	Permease activity as a function of outside glucose concentration.
*k*_*M*_	7.0 ×10^5^ *m* *p* *b*	Max. transcription initiation rate of lac promoter

The rate of lactose influx per permease (*p*) is set to *p*_*lac*_=1000 lactoses per *Δ* *T* per permease, from which we calculate the number of lactoses transported into the cytoplasm per *Δ* *T* as [5]:

(3) $$ L_{trans} = p_{lac} \times p.   $$

From the above equations, we can update the total number of lactoses *L* in the cytoplasm:

(4) $$ L := L - L_{mtbol} + L_{trans}.   $$

The lactose counts feed into the particle systems (described below) to visualize these entities in realtime, while keeping the simulation interactive (Fig. 1). A visual representation of lactose metabolism by *β*-galactosidase is depicted in Fig. 4. In order to simulate lactose transport through transmembrane permeases, an agent-based representation of this process has been implemented as well (Fig. 3).

### Visualizing a crowded cell space

A bacterial cell is filled with billions of water molecules, proteins, and other macro and micro molecules. Due to the critical role of proteins, each protein agent in the simulation is modeled as an individual physical entity. This means the agent has physical properties and interacts with its environment through physical collisions that trigger reactions and state changes in the system. Simulating physical interactions is computationally very expensive, though. Even with today’s powerful computers, their high computational capabilities (e.g., by utilizing GPU algorithms) and despite sophisticated physics simulation libraries (e.g., Bullet [14]), only a relatively small subset of participating entities can be considered for a physical simulation. However, only a very small subset of interacting molecules and proteins actually have direct effects on the lactose operon. In other models, we have used an ‘abstraction’ mechanism to alleviate the computational challenges with large, multi-agent simulations [41, 50]. The basic idea is to observe agent dynamics, extract interaction patterns, and temporarily replace groups of agents by a single ‘cluster agent’. The validity of this ’abstraction’ needs to be checked on a regular basis and, if necessary, all agents of a cluster will be released back into the simulation. In *Prokaryo*, however, we use yet another technique to visualize large numbers of entities (Fig. 1): particle systems, which we explain in the next section.

### Particle systems

In addition to proteins, other entities that protein agents interact with need to appear in large quantities (e.g., lactose). To simulate the crowded universe of biomolecules within a cell, we use a computer graphics technique known as *particle systems* [51]. Particle systems harness the power of graphics processors (GPUs) and are able to render small entities (particles) in large numbers. Particle systems have been used to simulate phenomena that consist of very small interacting elements such as water, sparks, clouds, fog or snow. With particle systems, we are able to visualize over 70,000 entities inside the cell, together with about 1,000 protein agents with actual physical properties, while maintaining full interactive control of the simulation.

## Results and discussion

Now that we have discussed the mathematical, computational, and rendering aspects of our modeling framework, we present a step-by-step description of our *Prokaryo* simulation. We explain which aspects are captured by our model and how the generated outcomes match the actual biomolecular processes inside an *E. coli* cell.

*Repressor*

**Fig. 16:**
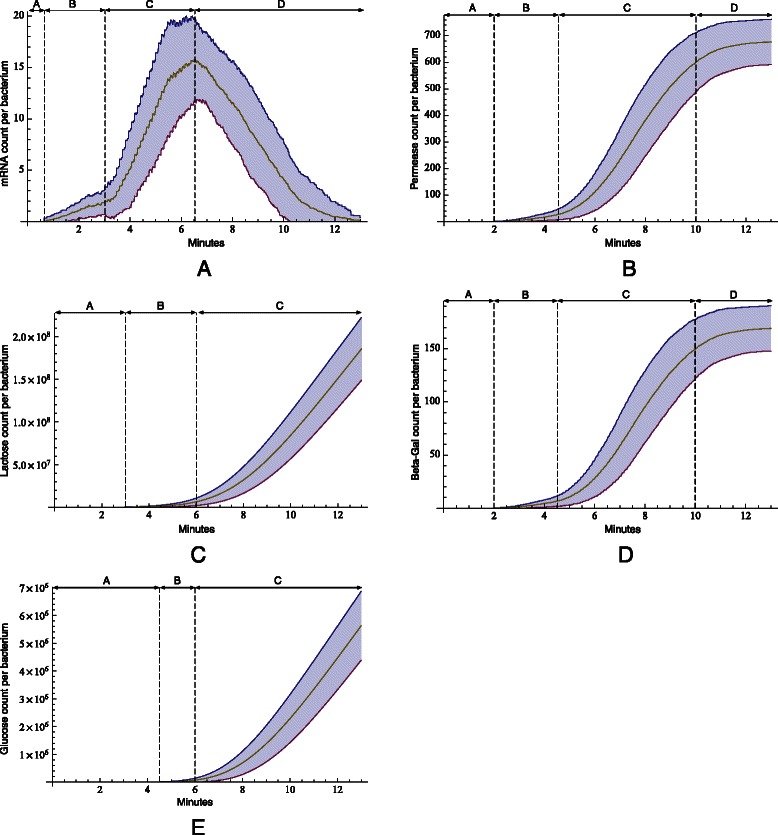
Simulation results from the *Prokaryo* model. mRNA counts from simulations over 30 trials. The shaded areas represent the standard deviation from the average counts

At the beginning of the simulation, the cell contains no glucose. Lactose is located outside the cell, with some lactose undergoing passive transport into the cell. As lactose concentration is below the *Rlt* threshold (Table 3), repressor is docked on the promoter, thus blocking transcription of DNA (Fig. 6). Each repressor undocks with probability *λ*_*rep*_. Consequently, multiple repressors are docking and undocking continuously. Between the undocking of one repressor and docking of another repressor, RNA polymerases may have a window of opportunity to attach to the promoter and initiate transcription with probability *λ*_*poly*_=1/100. For every 100 transcription initiation attempts, only one permease will be successful. This value was determined through a number of simulation experiments. A lower transcription initiation rate would cause our system to halt. A higher number would result in RNA polymerases to initiate transcription and ignore the presence of repressor. Figure 16 a illustrates the number of mRNAs resulting from transcription through polymerases. The basal level expression is labeled as Area B.

*Transcription and translation*

Unsuccessful attempts of transcription happen during the period marked as Area A. Once the operon is expressed at basal levels, resulting in mRNA copies, translation of permease and *β*-galactosidase is performed by ribosomes (Fig. 7). Permease acts as a transmembrane protein and actively transports lactose at a much higher rate than through passive transport alone. *β*-galactosidase metabolizes lactose into glucose and allolactose. Allolactose is subsequently metabolized into glucose and galactose. Allolactose deactivates the repressor, which detaches from the operator. The operon genes are now expressed at their highest level (Area C). The number of mRNAs triples in less than 4 minutes (real time). Once the system has reached the glucose threshold of 40,000 cpb, cAMP concentrations decrease with CAP no longer bound to DNA. This leads to a reduction in the rate of transcription (Area D).

*Lactose permease*

Figure 16 b illustrates lactose permease counts over time. Area A represents the initial 2 minutes of unsuccessful transcription attempts by RNA polymerase. During base level expression (Area B), permeases are being expressed, starting to initiate lactose transport into the cell. In Area C, the operon is expressed fully, producing over 600 permeases within about 5 minutes. An equilibrium^6^ is reached once lactose production has stopped due to the absence of CAP complex on the DNA (Area D).

*Lactose*

It takes about 3 minutes until lactose starts to get imported into the cell (Fig. 16 c, Area A). At base level expression, few active transmembrane proteins transport lactose into the cell, which initiates gene expression (Area B) via allolactose production. Once repressor is deactivated by allolactose, the lac operon genes are expressed at high levels. A rise in permease production leads to an exponential increase of lactose levels inside the cell (Area C). Even after gene expression decreases, the lactose count within the cell does not decrease. This is due to active permeases still transporting lactose into the cell.

*β* *-galactosidase*

It takes about 2 minutes before *β*-galactosidase is generated (Fig. 16 d). Base level expression leads to small production of *β*-galactosidase (Area B). Expression of lacY results in an increase in *β*-galactosidase (Area C). After the system reaches the glucose threshold, gene expression is minimized, thus halting production of *β*-galactosidase (Area D). The glucose threshold is set at 40,000 per bacterium. If the glucose threshold is considerably below this value, the lac operon switch is turned off too early. A higher threshold, causes lactose metabolism and transport to increase drastically.

The lac operon facilitates the process to metabolize lactose when glucose levels are low. Glucose is the desired product, which requires a complex, multiple step process to produce. The time required by the lac operon to switch and initiate gene expression for glucose production is depicted in Fig. 16 e (Area A). Base level expression results in a slight increase in the production of glucose (Area B). Once gene expression is activated by absence of repressor from the DNA, multiple polymerases transcribe the operon genes, which results in an increased count of mRNA. Consequently, more permease and *β*-galactosidase are produced (Fig. 16 a and b). Permease transports more lactose into the cell. More *β*-galactosidase is expressed from the operon. This results in an exponential increase in lactose metabolism and, in turn, in increased glucose production (Area C).

*Summary*

The hybrid approach produces results that are in good alignment with the results from the continuous Santillan model [27]. Comparing mRNA predicted by the differential equations and the hybrid model, the trend of mRNA generation and degradation is very similar (Figs. 9 a and 16 a). On average, 700 permeases are generated to transport lactose into the cell as shown by both the differential equations and the hybrid model (Figs. 9 b and 16 b).

Both models predict the same rate of lactose metabolism (in orders of 10^8^) as illustrated in Figs. 9 d and 16 c.

## Conclusion

We have presented a hybrid model of *E. coli* metabolism related to the lactose operon. The dynamics of cellular ‘agents’ are defined by their rules of interaction, implemented as finite state machines. The agents are embedded within a 3-dimensional virtual environment with simulated physical and electrochemical properties. The hybrid model is driven by a combination of (1) mathematical equations (DEQs) to capture higher-scale phenomena and (2) agent-based rules to implement localized interactions among a small number of molecular elements. By reproducing results from *in silico* experiments reported in the literature, we have demonstrated that our hybrid model is able to capture phenomena across multiple spatial scales, from changing concentration gradients to one-on-one molecular interactions.

**Fig. 17:**
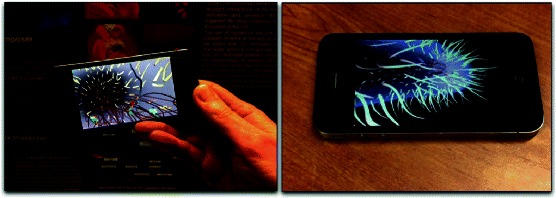
Application on mobile devices. *Prokaryo* running on an iPhone, making use of the touch interface for easy navigation

Our article also demonstrates how interactivity and illustrations, in the form of detailed graphical representations in 3-dimensional scenes, can enhance the understanding of biological systems through exploration and visualization. We have implemented our *Prokaryo* model on desktop computers, but also on mobile devices with touch interfaces (Fig. 17). Mobile computing devices make such models more accessible. Touch interfaces greatly facilitate navigation through and exploration of such models.

*Prokaryo* is only our first 3D, interactive model of a larger biomolecular system. We will extend *Prokaryo* by including other pathways (such as chemotaxis) and embed *Prokaryo* into a colony of interacting cells. *Prokaryo* is part of the *Lindsay Virtual Human* (*LINDSAY*), a multi-scale computational physiology simulation environment [38], in which we can place *E. coli* cells inside the *LINDSAY* intestinal tract. Any cell can be selected, and one can navigate into its ‘cellular universe’, which is what we have described here as *Prokaryo*. More information on the Lindsay Virtual Human can be found at http://lindsayvirtualhuman.org, including a video demonstration and executable download (http://www.lindsayvirtualhuman.com/?page_id=469).

## Endnotes

^1^ Neither of these game development engines had been available at the time we started this project. In our most recent simulation projects we use both game engines to build our simulations.

^2^ Physics engines are used extensively in computer games to simulate physical properties of 2- and 3-dimensional elements. Checking whether elements have collided and how such a physical interaction affects the colliding elements is implemented through physics engines.

^3^ In our model, *Δ* *T*=1/60 second is the length of a simulation step as defined by the physics engine [14].

^4^ We are aware that folding should occur earlier, but we have ignored this aspect to simplify our current model.

^5^ Similar schemes for hybrid models have been developed recently. Most noteworthy is a hybrid model of bacterial ecology and chemotaxis [52]. We roughly follow the hybrid modeling framework proposed in this work. Compare, for example, Fig. 1 in [52] and Fig. 10.

^6^ The permease count remains constant due to a permease degradation rate of zero.
